# Compartmental modeling of whole-body vitamin A kinetics in unsupplemented and vitamin A-retinoic acid-supplemented neonatal rats[S]

**DOI:** 10.1194/jlr.M050518

**Published:** 2014-08

**Authors:** Libo Tan, Amanda E. Wray, Michael H. Green, A. Catharine Ross

**Affiliations:** * Graduate Program in Nutrition, The Pennsylvania State University, University Park, PA 16802; † Department of Nutritional Sciences, The Pennsylvania State University, University Park, PA 16802; § The Huck Institutes for the Life Sciences, The Pennsylvania State University, University Park, PA 16802

**Keywords:** neonate, retinol, chylomicrons, extrahepatic tissues, Windows version of the Simulation, Analysis, and Modeling software

## Abstract

Little is known about the contribution of different tissues to whole-body vitamin A (VA) kinetics in neonates. Here, we have used model-based compartmental analysis of tissue tracer kinetic data from unsupplemented (control) and VA-retinoic acid (VARA)-supplemented neonatal rats to determine VA kinetics in specific tissues under control and supplemented conditions. First, compartmental models for retinol kinetics were developed for individual tissues, and then an integrated compartmental model incorporating all tissues was developed for both groups. The models predicted that 52% of chylomicron (CM) retinyl ester was cleared by liver in control pups versus 22% in VARA-treated pups, whereas about 51% of VA was predicted to be extrahepatic in 4- to 6-day-old unsupplemented neonatal rats. VARA increased CM retinyl ester uptake by lung, carcass, and intestine; decreased the release into plasma of retinol that had been cleared by liver and lung as CM retinyl esters; stimulated the uptake of retinol from plasma holo-retinol binding protein into carcass; and decreased the retinol turnover out of the liver. Overall, neonatal VA trafficking differed from that previously described for adult animals, with a larger contribution of extrahepatic tissues to CM clearance, especially after VA supplementation, and a significant amount of VA distributed in extrahepatic tissues.

Vitamin A (VA) plays critical roles in prenatal and perinatal development (1–4), yet little is currently known about VA metabolism in the neonatal period. To begin to fill this gap, we recently reported models for retinol kinetics in neonatal rats, based on plasma tracer data obtained for 14 days after an oral dose of [^3^H]retinol given to neonates on postnatal day (pnd) 4 (5). Such models of retinol kinetics as “viewed from the plasma space” are a necessary step both for developing more complex models that describe VA metabolism in specific tissues and as a guide for potential studies in humans, in which tissues cannot be sampled. For our modeling studies, we utilized [^3^H]retinol as a tracer for two treatments, an oil (control placebo) treatment in which we determined VA kinetics in unsupplemented neonates and a VA-retinoic acid (VARA) treatment in which the neonates received VA, given at a dose that resembles, adjusted for body weight, the amount of VA given to young infants in VA supplementation studies (6). This dose was admixed with one-tenth the amount of RA, a combination we have shown previously to promote retinol uptake into lung tissue (7, 8). The oil and VARA doses, each of which contained [^3^H]retinol, were administered orally to pnd 4 neonatal rats, and plasma was monitored until pnd 18. From this plasma view model, information on the transfer, turnover, storage, and disposal of retinol, as well as on the effects of VARA supplementation on VA kinetic behavior in neonatal rats, was obtained. The model predicted more extensive recycling of retinol between plasma and tissues in neonates compared with that in adults (9). VARA supplementation decreased the recycling number for retinol between plasma and tissues and the time that retinol spends in plasma; it also stimulated the uptake of plasma VA into extravascular tissues (5).

A more detailed view of retinol kinetics is possible when, in addition to plasma, the content of [^3^H]retinol in tissues is monitored over time. In the studies mentioned previously, we collected not only plasma but also various organs and the remaining carcass at each sample time point. By subjecting these tissues to tracer quantification, it is possible to develop a more detailed compartmental model for retinol kinetics that provides information on both whole-body and organ-specific dynamics, and thus a more complete picture of whole-body VA metabolism.

The contribution of different tissues to whole-body VA metabolism and kinetics under various conditions has been well explored in adult rats (9–17). For example, it has been determined that the liver plays the most critical role in VA homeostasis, as it is the primary site of chylomicron (CM) remnant uptake, clearing about 75% of newly absorbed VA (10, 11), and also, under VA adequate conditions, it is the major site of VA storage (17). In adult rats, ∼25% of dietary retinyl esters are taken up by extrahepatic tissues, including adipose tissue, skeletal muscle, heart, lung, and kidney (10, 12). Extrahepatic tissues that are active in metabolizing CM triglyceride, such as adipose tissue and the mammary gland during lactation, may acquire newly absorbed VA during lipolysis of CM triglycerides by LPL (12, 13). Regarding retinoid supplementation, a previous modeling study (14) showed that chronic dietary administration of the VA metabolite retinoic acid (RA) to rats with low VA status affected the recycling, uptake, and mass of VA in a tissue-specific manner. Specifically, RA supplementation increased the total time that VA spends in liver, kidney, small intestine, and lung, and it increased the total VA mass within these tissues, as compared with rats fed the same diet without RA.

Unlike for adult rats, the contribution of different tissues to whole-body VA kinetics, distribution of whole-body VA, and the trafficking of VA between plasma and organs have not yet been studied in neonates, although it has been suggested that in neonatal rats the lungs depend, at least in part, on the uptake of dietary VA, probably from CM, to develop retinyl ester stores in the postweaning growth period (18). Building on the plasma view models recently proposed (5), in the present study we have developed compartmental models for individual tissues, including liver, lung, stomach, intestine, kidney, and the remaining carcass, and an integrated model for control and VARA-treated neonatal rats that incorporates all of these tissues. This analysis provides new insight into VA kinetics at both a whole-body and organ-specific level as well as on the effect of VARA on the kinetic behavior of retinol in neonatal rats.

## MATERIALS AND METHODS

### Kinetic study and analytical procedures

Details of the design and conduct of the kinetic study have been presented previously (5) and therefore are described here in brief.

### Animal and diets

Animal protocols were approved by the Pennsylvania State University Institutional Animal Care and Use Committee. After mating, Sprague-Dawley female rats were fed a diet containing a marginal level of VA (0.35 mg retinol equivalents/kg diet) throughout the study period. Rat pups were assigned randomly to two treatments, a control (oil) group given canola oil as placebo and a VARA treatment group.

### Preparation of the oral dose

The amount of VA (*all-trans* retinyl palmitate) in the oral VARA dose [∼6 mg/kg body weight (7)] was based on the amount of VA administered to human infants to reduce morbidity and mortality (50,000 IU/∼2.5 kg body weight) (6). VARA was a mixture of the VA dose and one-tenth the amount of RA [∼0.6 mg/kg body weight (7)]. The VARA mixture, or oil only, was admixed with 11,12-[^3^H]retinol as described previously (5). The radioactivity in the oral dose was 0.2 µCi/µl.

### Kinetic study

On pnd 4, each pup received a single oral dose containing 11,12-[^3^H]retinol admixed with either oil or VARA. The dose, about 1.8 µCi/neonate, was delivered directly into the mouth via a small micropipette. Aliquots of each dose preparation were extracted and analyzed for [^3^H]retinol to determine the exact value for 100% of the dose for each neonate (5). Immediately after dosing, pups were returned to their mothers. Groups of pups (*n* = 3 per time per group) were euthanized with isoflurane at 14 time points after dosing: 1, 2.5, 4, 6, 8, 11, 15, and 24 h; and 2, 4, 6, 8, 11, and 14 days. Blood was collected from the vena cava into heparinized syringes. Liver, lung, stomach, intestine, kidney, thymus, spleen, heart, and the remaining carcass were excised and rapidly frozen in liquid nitrogen.

### Tissue analysis

Portions of tissue were weighed and homogenized in 100% ethanol using a glass homogenizer. Tissues were incubated in ethanol for 1 h, and then lipids were extracted with hexanes containing 0.1% butylated hydroxytoluene (BHT). After centrifugation to separate the organic and aqueous phases, the upper hexane phase was removed into new vials, and solvent was evaporated under argon. The extraction with hexane/BHT was repeated. For liver and lung tissues, a measured portion of the extract was taken for analysis of radioactivity by liquid scintillation spectrometry, using Scintiverse (Fisher Scientific) as liquid scintillation fluid. For other tissues, all of the extract was evaporated to dryness and analyzed for ^3^H radioactivity by liquid scintillation spectrometry. Samples were counted to a 1% counting error (19).

### Calculation of the fraction of the oral dose

The fraction of the ingested dose remaining in a given tissue at each sampling time for each neonate was calculated as total radioactivity (dpm) in that tissue divided by dpm in the ingested dose for that pup. Radioactivity in the ingested dose was calculated as the dose total dpm minus the dpm remaining in the pipette tip and paper chip (used to blot the muzzle of pups that had any visible oil on them) combined.

### Model development

Model-based compartmental analysis was applied to the tracer response profiles (the mean fraction of ingested dose vs. time after dose administration) from both groups using Windows version of the Simulation, Analysis, and Modeling software (WinSAAM) (20, 21). To develop compartmental models for individual tissues and the subsequent integrated model that incorporates all tissues, an option in WinSAAM called the “forcing function” was applied, similar to the method described by Cifelli et al. (14). The forcing function approach allows the modeler to develop initial models for each organ before working with all organs simultaneously. This approach makes use of the fact that plasma is the sole source of VA input to individual organs or tissues, except for stomach and intestine. Because each organ exchanges VA directly with plasma but not with other organs, individual organs can be uncoupled from the whole system and modeled individually based on the plasma tracer response profile and tracer data from the organ. For the former, we used model-simulated plasma tracer response profiles from our plasma view model (5), details of which are presented in supplementary Fig. I. When developing models for individual tissues, these simulated plasma tracer responses were applied as a “QL” function (20) in WinSAAM, which is used for generation of responses from data. In our modeling, the plasma forcing function was applied to each organ to develop an individual organ model. The set of these forcing function models will be called “model 1” in this paper. The process of model development and model fitting was similar to that for our published plasma-view model (5). First, hypothesized models (shown in Results) were set up, and then tissue tracer data were analyzed in light of the proposed model using WinSAAM until a satisfactory fit was obtained between observed and model-predicted values. Specifically, model parameters (fractional transfer coefficients; see below) and, when necessary, model structure were iteratively adjusted until a close fit was obtained as judged by visual inspection of the simulated tracer data plot and by statistical analysis, including the sum of squares from nonlinear regression analysis and the estimated fractional standard deviation (FSD) for each kinetic parameter. An F statistic (22) and the Akaike information criterion [AIC (23)] were used to statistically test whether increases in model complexity were justified. The model complexity was increased only when it resulted in a significant improvement in the sum of squares as determined by an F statistic and reduced AIC by more than 1–2 units. Once a satisfactory fit was achieved, the final estimates of the fractional transfer coefficients [L(I,J)s] and their statistical uncertainties were generated by weighed nonlinear regression analysis in WinSAAM. For weighting purposes, an FSD of 0.05 was assigned to each datum of tissue kinetics. Parameters were considered well identified if their estimated variability (FSD) was <0.5.

Once data for all organs were satisfactorily fit, individual organ models for each group were combined into an integrated model, referred to as “model 2.” The forcing function was released, and the entire data set, including the observed kinetic data for plasma and all other tissues, was modeled simultaneously to obtain the integrated model. When a satisfactory fit was achieved, final estimates of the fractional transfer coefficients [L(I,J)s; see below] and their statistical uncertainties were obtained. More details on releasing the forcing function and the development of model 2 are presented in Results.

### Model-predicted kinetic parameters

The fractional transfer coefficient [L(I,J)] is the fraction of retinol in compartment J that is transferred to compartment I each day; thus L(I,J)s are parameters that define the behavior of the system. Traced mass [M(I)] is the model-predicted amount of retinol in compartment I during a steady state. M(I)s were calculated for extravascular compartments using the plasma retinol pool sizes in a steady-state solution in WinSAAM. Plasma retinol pool sizes were estimated as the product of plasma retinol concentration obtained from UPLC analysis of plasma (5) and the plasma volume, estimated as 3.5% of body weight. Parameters were compared between neonates in the control group and the VARA group.

### Statistical analysis

Compartmental modeling was done using group mean data at each time [this represents a “super-pup” model, analogous to a previous “super-rat” model (14) and to approaches used in other modeling (24)]. For kinetic parameters, L(I,J)s are presented with estimated FSDs. Differences in L(I,J)s between groups, *P* < 0.05, were determined by using an unpaired *t*-test. Briefly, a *t* statistic for each L(I,J) was calculated using the equation (Value_1_ − Value_2_)/sqrt(SEM_1_^2^ + SEM_2_^2^), which was then evaluated for significance using a table of *t* values.

## RESULTS

### Kinetics of tissue VA

**Fig. 1.**
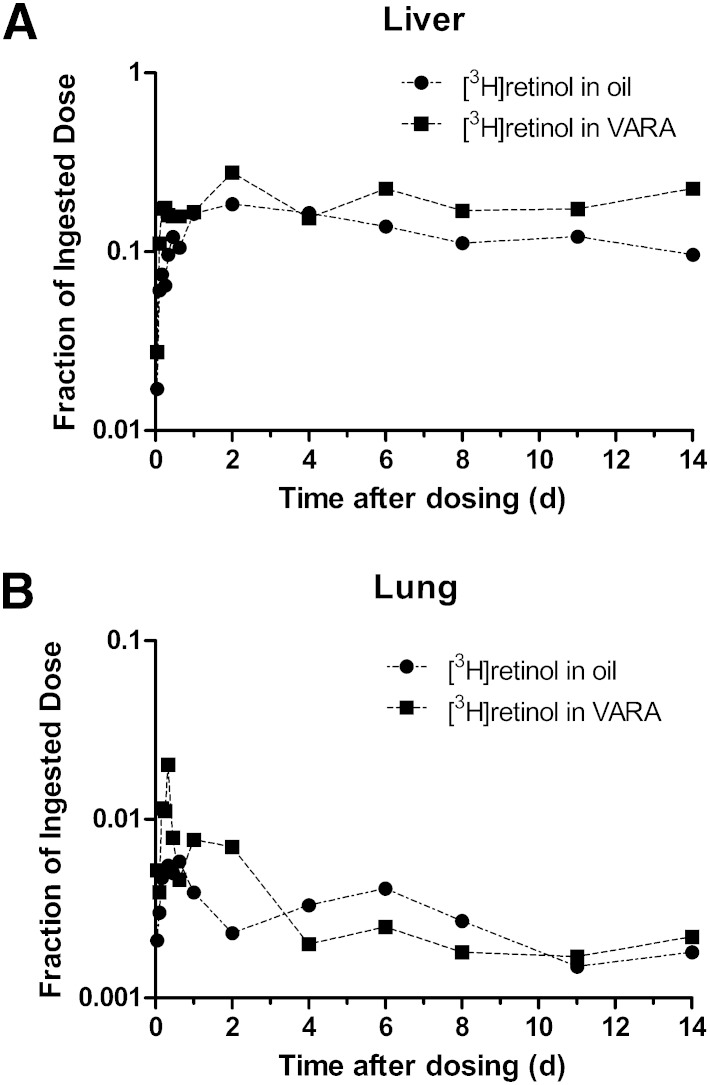
Mean observed fraction of administered dose in liver (A) and lung (B) versus time (days) after administration of [^3^H]retinol in oil or in VARA to neonatal rats. Each point represents the mean of *n* = 3 pups.

Data on fraction of the dose remaining in liver and lung versus time after dose administration are plotted semilogarithmically in **Fig. 1**. In liver (Fig. 1A), radioactivity increased until 2 days after dosing in both groups. The maximum recovery of radioactivity from liver was 18% in control pups and 28% in VARA-treated pups. After 2 days postdosing, radioactivity in control neonates decreased gradually until the end of the study, while the fraction of dose remained relatively constant in VARA-treated neonates. Except for values 4 days postdosing, the mean fraction of the dose in liver was higher in VARA-treated pups than in control pups.

In lung (Fig. 1B), the percentage of dose was <1% at most time points. Radioactivity rose within the first several hours and reached a peak 8 h after dose administration in both groups. The percentage of dose at the peak in the control group was 0.55%, whereas it reached 2% in the VARA group. After the peak, radioactivity decreased rapidly and remained relatively constant in both groups.

**Fig. 2.**
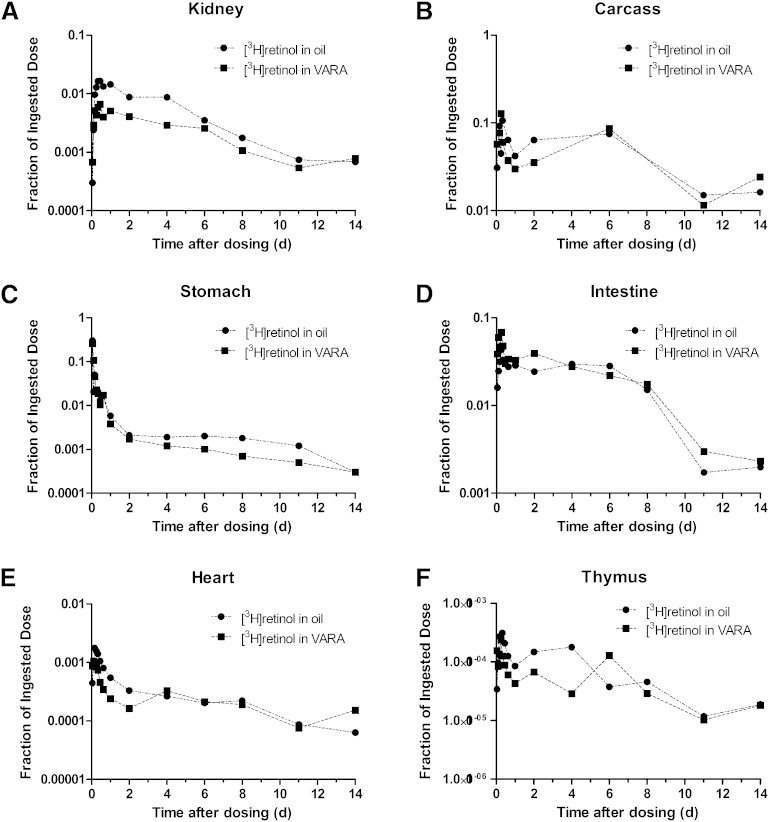
Mean observed fraction of administered dose in kidney (A), carcass (B), stomach (C), intestine (D), heart (E), and thymus (F) versus time (days) after administration of [^3^H]retinol in oil or in VARA to neonatal rats. Each point represents the mean of *n* = 3 pups.

Data on fraction of the oral dose remaining in kidney, carcass, stomach, intestine, thymus, and heart versus time after dose administration are plotted in **Fig. 2**. The fraction of the dose in kidney (Fig. 2A) in the control neonates was higher than that in the VARA-treated neonates at most time points; however, the pattern of the kinetic response was similar between groups. A rise in radioactivity was observed within 1–11 h after dose administration, and then it decreased until the end of the study. The remaining carcass (Fig. 2B), which was mainly composed of skin, bone, muscle, brain, and adipose tissue, contained the second highest fraction of the ingested dose, after liver. In both groups, radioactivity rose during the first several hours to a peak at 4–6 h where the fraction of dose was ∼10% to 12%. Following a rapid decrease after the peak, there was an increase in the fraction of dose from 1 day to 6 days after dose administration. After day 6, the fraction of dose in the carcass decreased in both groups.

About 35% of the tracer was found in the stomach (Fig. 2C) 1 h after dose administration in both groups. Tracer disappeared rapidly from the stomach. After 2 d, the decrease became more gradual, which might indicate recycling of retinol between plasma and stomach. For the intestine (Fig. 2D), a rise in radioactivity was observed in the first several hours after dose administration in both groups, followed by a decrease and then a plateau period from 1 day to 6 days in both groups. After 8 days, radioactivity decreased dramatically.

The fraction of the dose in heart, thymus (Fig. 2E, F), and spleen [see (25)] was low, ≤0.1% at all times, and the patterns of tracer response in these tissues were similar in that radioactivity peaked within the first several hours after dose administration, and by day 2, the [^3^H]retinol decayed and then remained low. The appearance of [^3^H]retinol in spleen and thymus indicates that orally administered VA is taken up to some extent by immune tissues. Although the percentage of [^3^H]retinol was low at all times, a definite kinetic pattern was observed in each tissue.

Overall, the data in Fig. 1 and Fig. 2 define the tissue kinetic responses for individual organs to an oral dose of [^3^H]retinol and indicate that each tissue has a characteristic pattern of metabolism (retinol trafficking) during the initial absorptive phase and after the absorptive period has been completed.

### Development of the forcing function model (model 1)

Next, we developed a model for each organ subsystem, based on tracer data for that organ and using the forcing function option in WinSAAM. For this, we used the model-simulated plasma tracer response profiles (supplementary Fig. I) from our plasma-view model (5), which was based only on fitting the observed plasma kinetic data. The profiles indicated that the tracer almost disappeared from CM retinyl esters during day 1 in the control group and by 8 h in the VARA group (supplementary Fig. IA); radioactivity rose rapidly in plasma retinol in the first several hours after dosing (supplementary Fig. IB).

**Fig. 3.**
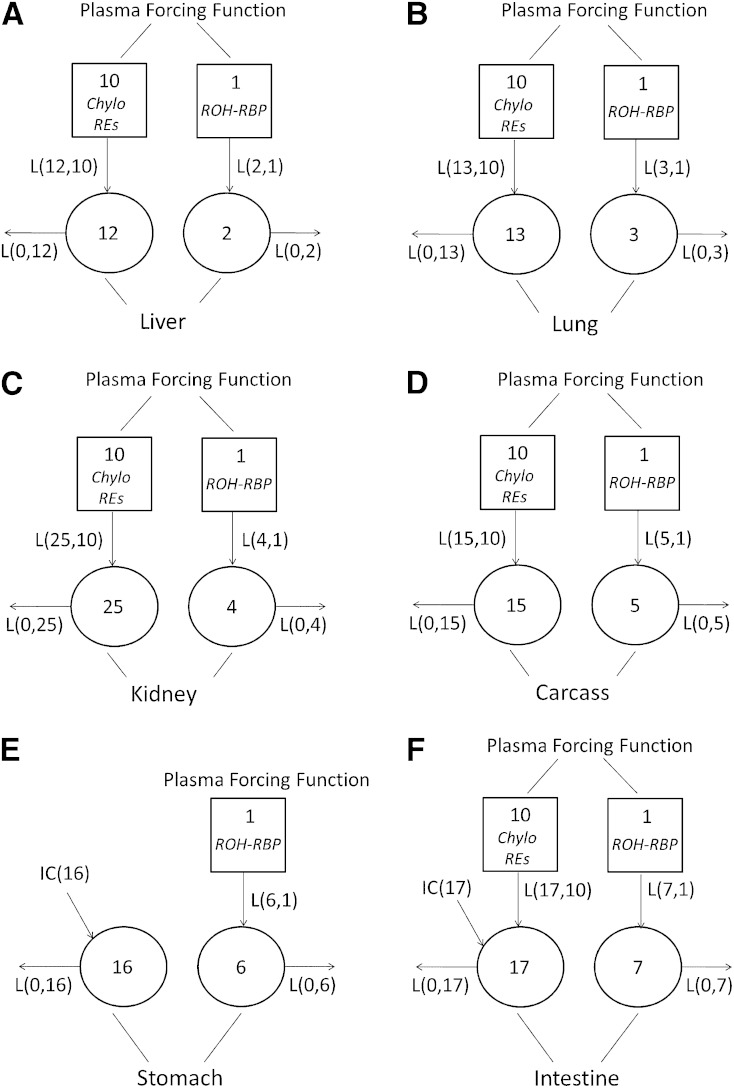
Proposed models with forcing function for VA metabolism (model 1) in liver (A), lung (B), kidney (C), carcass (D), stomach (E), and intestine (F) in neonatal rats administered [^3^H]retinol in oil or in VARA based on the tissue tracer response data. Compartments are represented as circles, and interconnectivities between compartments correspond to L(I,J)s or the fraction of the material in compartment J transferred to compartment I per day. The squares represent plasma forcing functions. Compartment 10 describes the profile of plasma tracer in CM/CM remnants retinyl ester. Compartment 1 describes the profile of plasma tracer in retinol. Compartments 12, 13, 29, and 15 represent retinyl ester that is taken up from plasma CM/CM remnants by liver, lung, kidney, and carcass, respectively. Compartments 2, 3, 4, 5, 6, and 7 represent VA in liver, lung, kidney, carcass, stomach, and intestine, respectively, that exchanges with plasma retinol. Compartment 16 represents the processing of newly ingested VA in stomach. Compartment 17 represents the processing of newly ingested VA in intestine and retinyl ester that is taken up from plasma CM/CM remnants by intestine. IC(16) and IC(17) represent the newly ingested dose that enters the stomach and the intestine, respectively.

Compartmental model 1 with the forcing function applied, shown in **Fig. 3**, is composed of six individual models for neonatal liver, lung, kidney, carcass, stomach, and intestine. Because the fraction of ingested dose found in thymus, heart, and spleen was very low throughout the study period, tracer data for these three tissues were incorporated into that of the carcass for the purpose of model development. Models established for liver, lung, kidney, and carcass (Fig. 3A–D) are similar. For these tissues, plasma is the sole source of VA input. Models were developed based on the assumption that, because the dose was given orally, there are two possible inputs that affect VA kinetics in each tissue: the uptake of retinyl ester, likely in plasma CM/CM remnants by tissues, and the turnover of retinol, >95% of which is bound to retinol binding protein (RBP) (26) in plasma, between tissues and plasma. The model developed for liver (Fig. 3A) will be presented as an example. Compartment 12 represents retinyl ester that is taken up by the liver from plasma CM remnants (compartment 10), and compartment 2 represents liver VA that exchanges with plasma retinol (compartment 1). The tracer data for liver were assigned to compartment 12 plus compartment 2. Because the forcing function was applied, compartments 10 and 1, which represent CM retinyl ester and plasma retinol, respectively, were included in the model to indicate the source of VA for the liver. L(12,10) (Fig. 2A) represents the fraction of retinyl ester that is cleared from CM remnants by liver each day. L(2,1) is the fraction of retinol that is transferred from plasma into liver each day. These models do not show any recycling of retinol back to plasma because the plasma tracer response is fixed as input when the forcing function is applied. As a result, L(0,12), the fraction of retinol that entered from CM retinyl ester and leaves the liver each day, includes the fraction of retinol that is secreted from liver into plasma after processing. L(0,2), the fraction of retinol that leaves the liver each day, includes the irreversible loss of retinol from liver and the fraction that recycles back to plasma.

Models developed for stomach and intestine (Fig. 3E, F) differ from those for liver, lung, kidney, and carcass because plasma is not the sole source of VA for those tissues. In the model for stomach, there are two compartments: compartment 16 represents the processing of newly ingested VA in stomach, and compartment 6 is the VA in stomach that exchanges with plasma retinol (compartment 1). An initial condition (IC), shown as IC(16) in Fig. 3E, was included in the model to represent the newly ingested dose that enters the stomach. The model for the intestine also includes two compartments, 17 and 7. Compartment 17 represents the processing of newly ingested VA in the intestine, and 7 is the intestinal VA that exchanges with plasma retinol (compartment 1). IC(17) in Fig. 3F was included to represent the newly ingested dose that enters the intestine from the stomach. We found that, to fit the intestine tracer data, an input from compartment 10 [L(17,10)] was necessary, suggesting that there is some uptake of plasma CM retinyl ester by the intestine.

**Fig. 4.**
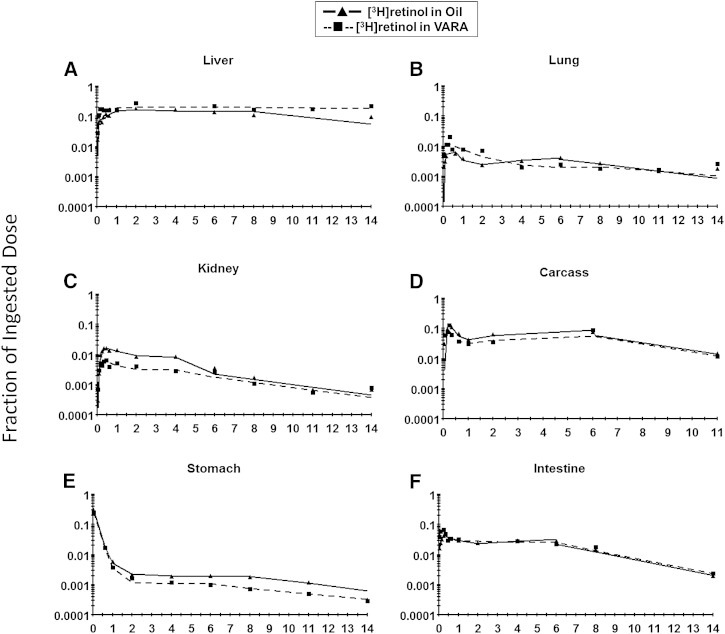
Mean observed (symbols) and model 1-predicted fraction of administered dose (lines) in liver (A), lung (B), kidney (C), carcass (D), stomach (E), and intestine (F) versus time (days) after administration of [^3^H]retinol in oil or in VARA to neonatal rats. Each point represents the mean of *n* = 3 pups.

The observed and model-predicted tracer responses in different tissues in both the control group and the VARA group are shown in **Fig. 4**. The fit of the proposed models to the data can be seen by comparing the observed data (points) and the model-predicted values (smoothed lines).

### Model 1-derived kinetic parameters

The estimates of fractional transfer coefficients [L(I,J)s] and FSDs for both groups obtained from model 1 are shown in **Table 1**. Parameters were well defined with FSDs of 0.03–0.15. L(13,10), L(15,10), and L(17,10), which represent the fraction of plasma retinyl ester that is taken up by lung, carcass, and intestine, respectively, were significantly (*P* < 0.01) increased in VARA-treated pups. The fraction that was taken up by carcass was increased ∼5 times by VARA. L(12,10), the fraction of plasma retinyl ester in CM remnants that is cleared by liver, was also significantly (*P* < 0.05) increased by VARA. L(0,12), L(0,13), L(0,25), and L(0,15), which represent the fraction of the retinol tracer dose that leaves the liver, lung, kidney, and carcass each day, respectively, were decreased greatly (*P* < 0.01) by the VARA treatment. L(0,12), the fraction of CM remnant-derived retinol leaving the liver each day, was almost 0 in the VARA group. The VARA treatment increased the uptake of plasma retinyl ester into tissues, especially carcass, while it decreased the fraction of retinol leaving the tissues after being processed. L(5,1), the fraction of retinol that is transferred from plasma into carcass each day, was significantly (*P* < 0.05) increased by VARA, while L(0,2), the fraction of retinol turnover out of the liver each day, was greatly decreased by VARA (*P* < 0.01).

**TABLE 1.** The fractional transfer coefficients predicted by the forcing function model (model 1)

L(I,J)	Tissue	Treatment Group	
		Oil	VARA
		Value (day^−1^; FSD)	
L(12,10)	Liver	168 (0.13)	223 (0.06)*^a^*
L(0,12)		50.0 (0.15)	0.25 (0.58)*^a^*
L(2,1)		36.9 (0.05)	28.1 (0.13)
L(0,2)		1.20 (0.06)	0.38 (0.09)*^a^*
L(13,10)	Lung	2.68 (0.04)	32.6 (0.03)*^a^*
L(0,13)		4.07 (0.05)	0.70 (0.07)*^a^*
L(3,1)		0.21 (0.04)	0.14 (0.12)*^a^*
L(0,3)		0.16 (0.04)	0.11 (0.14)*^a^*
L(25,10)	Kidney	3.22 (0.03)	1.37 (0.39)*^a^*
L(0,25)		2.17 (0.12)	0.06 (2.00)*^a^*
L(4,1)		1.23 (0.15)	3.01 (0.09)*^a^*
L(0,4), before dosing day 8		0.71 (0.15)	2.98 (0.08)*^a^*
L(0,4), after dosing day 8		0.32 (0.06)	0.23 (0.40)
L(15,10)	Carcass	89.9 (0.09)	438 (0.05)*^a^*
L(0,15)		14.1 (0.11)	5.80 (0.07)*^a^*
L(5,1)		6.06 (0.04)	7.24 (0.04)*^a^*
L(0,5)		0.29 (0.04)	0.31 (0.03)
L(0,16)	Stomach	5.06 (0.03)	5.00 (0.05)
L(6,1)		1.50 (0.68)	0.57 (1.71)
L(0,6), before dosing day 8		3.72 (0.05)	1.37 (1.71)
L(0,6), after dosing day 8		3.72 (0.05)	0.14 (0.06)*^a^*
L(17,10)	Intestine	10.2 (0.07)	327 (0.15)*^a^*
L(0,17)		2.79 (0.09)	7.91 (0.14)*^a^*
L(7,1)		2.26 (0.04)	9.77 (0.10)*^a^*
L(0,7), before dosing day 8		0.29 (0.02)	1.00 (0.11)*^a^*
L(0,7), after dosing day 8		0.39 (0.05)	0.29 (0.03)*^a^*
IC(16)	Stomach	0.36 (0.05)	0.32 (0.05)
IC(17)	Intestine	0.02 (0.05)	0.00 (0.00)

The data are fractional transfer coefficients [L(I,J)s or the fraction of retinol in compartment J that is transferred to compartment I each day (estimated FSDs in parentheses)] predicted by the forcing function model (model 1; shown in Fig. 3), which is composed of six compartmental models developed for liver, lung, kidney, carcass, stomach, and intestine with forcing function applied. L(I,10)s represent the fraction of plasma retinyl ester in CM/CM remnants that is taken up/cleared by tissues each day. L(0,I)s [L(0,12), L(0,13), L(0,25), L(0,15), and L(0,17)] are the fraction of retinol that leaves tissues after being processed each day that came from CM. L(I,1)s are the fraction of retinol that is transferred from plasma into tissues each day. L(0,I)s [L(0,2), L(0,3), L(0,4), L(0,5), L(0,6), and L(0,7)] represent the fraction of recycled retinol that leaves tissues each day. IC(16) and IC(17) are the calculated fraction of the dose at *t*_0_ in stomach and intestine, respectively.

a Indicates significant differences (*P* < 0.05) from the control group.

### Plasma VA output to tissues

The percentages of CM retinyl ester taken up by different tissues and of plasma retinol transferred to different tissues were calculated from L(I,10)s and L(I,1)s, through which the contribution of different organs to plasma VA output could be estimated. **Table 2** shows the results for both groups. For oil-treated pups, ∼40% of plasma retinyl ester in CM was taken up by carcass, which mainly consists of skin, brain, bone, heart, muscle, and adipose tissue, before being cleared by the liver, while about 5% was taken up by intestine and ∼2% by kidney plus lung. The remaining 52% was cleared by liver. In VARA-treated pups, extrahepatic tissues, mainly carcass and intestine, played a more important role in taking up CM retinyl ester. Carcass took up ∼43% and intestine ∼32%, while liver cleared only 22% of CM retinyl ester. VARA had a dramatic stimulatory effect on the uptake of retinyl ester back into the intestine. For plasma retinol turnover, the highest percentage went to liver in both groups (76% in the control group and 56% in the VARA group). More than 10% of plasma retinol output was taken up by the carcass in both groups. The VARA treatment stimulated plasma retinol turnover to the intestine. Kidney took up a measurable percentage of retinol (3.3% in oil-treated pups and 6.3% in VARA-treated pups).

**TABLE 2.** Calculated percentage of plasma VA derived from CM retinyl ester and plasma retinol that goes to different tissues in neonatal rats

Plasma VA	Tissue	Percentage	
		Oil	VARA
Plasma retinyl ester in CM/CM remnant	Liver	52.3%	22.1%
	Carcass	40.3%	42.7%
	Intestine	4.68%	31.8%
	Kidney	1.49%	0.13%
	Lung	1.24%	3.17%
Plasma retinol	Liver	75.6%	56.2%
	Carcass	12.8%	15.3%
	Intestine	4.77%	20.5%
	Kidney	3.27%	6.34%
	Stomach	3.12%	1.37%
	Lung	0.46%	0.30%

The data are the percentages of plasma retinyl esters in CM/CM remnant and of plasma retinol, considered holo-RBP, that goes to different tissues, which were calculated from L(I,10)s and L(I,1)s (shown in Table 1) of the forcing function model (model 1). For example, L(12,10)/[L(12,10) + L(13,10) + L(25,10) + L(15,10) + L(16,10) + L(17,10)] × 100% is the percentage of plasma retinyl ester that is cleared by liver, and L(2,1)/[L(2,1) + L(3,1) + L(4,1) + L(5,1) + L(6,1) + L(7,1)] × 100% is the percentage of plasma retinol turnover that goes to liver.

### VA distribution in neonatal rats

Retinol masses in different compartments [M(I)s] can be calculated from the estimated plasma retinol pool size in a steady-state model solution in WinSAAM. Although neonatal rats in this experiment were growing during the 18-day study and thus under metabolic nonsteady-state conditions, we observed that pups’ body weights were relatively constant in the first 2 days after dose administration (5). In addition, in the control group, plasma total retinol concentration was relatively constant throughout the study (5). Therefore, we calculated the average plasma retinol pool size (0.413 nmol), and, assuming a “steady state” with regard to plasma retinol pool during the first 2 days, we used this value to estimate VA masses in other compartments for control neonates and thus the tissue distribution of VA in this group (**Table 3**). Due to the perturbation of the VARA treatment on tissue retinol concentration (5), a relatively “steady state” was not found, and therefore no comparable calculation was done for the VARA-treated group. The model for the oil (control) group predicted that liver contained the most retinol (12.8 nmol). The carcass had the second highest amount (8.6 nmol), followed by intestine, kidney, lung, and stomach. About 50.1% of whole-body VA was present in extrahepatic tissues. Measured retinol masses in liver and lung of oil-treated pups in the first 2 days after dosing as calculated from the analytical results obtained by UPLC are also shown in Table 3. These values agree well with the model-predicted values. Therefore, the model appears to be powerful in predicting tissue retinol masses.

**TABLE 3.** Percentage of traced masses in organs predicted by forcing function model (model 1) and measured total retinol mass in liver and lung of oil-treated neonatal rats in the first 2 days after [^3^H]retinol dose administration

M(I)	Tissue	Percentage (%)	Model-Predicted Value	Measured Total Retinol Mass in the Tissue
			nmol	nmol
M(2)	Liver	48.4%	12.7	15.0
M(3)	Lung	1.91%	0.50	0.35
M(4)	Kidney	2.66%	0.70	ND
M(5)	Carcass	32.8%	8.64	ND
M(6)	Stomach	0.63%	0.17	ND
M(7)	Intestine	12.0%	3.17	ND

The data are model-predicted retinol masses [M(I)s] in liver, lung, kidney, carcass, stomach, and intestine of [^3^H]retinol in oil-treated neonatal rats in the first 2 days after dose administration. Pups’ ages were from pnd 4 to 6 during this period. Traced masses were predicted by the forcing function model (model 1) after including the plasma retinol pool size [M(1)] obtained from UPLC results in a steady-state solution in WinSAAM. Percentage is calculated by M(I)/[M(1) + M(2) + M(3) + M(4) + M(5) + M(6) + M(7)] × 100%. ND, not determined.

### Proposed integrated compartmental model (model 2)

**Fig. 5.**
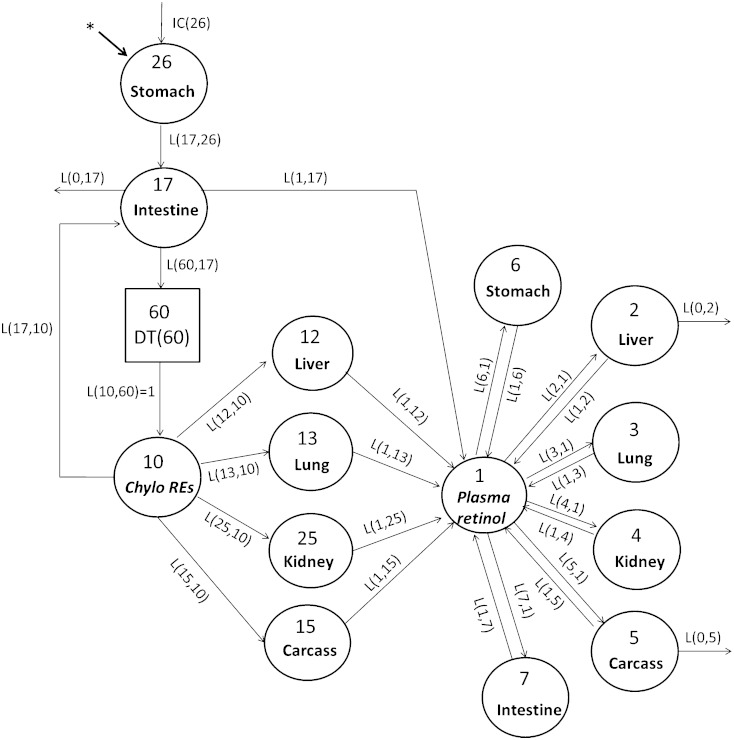
Proposed integrated compartmental model for VA metabolism (model 2) in neonatal rats administered [^3^H]retinol in oil or in VARA. Compartments are represented as circles and interconnectivities between compartments correspond to L(I,J)s or the fraction of the material in compartment J transferred to compartment I per day. Compartment 26 represents the processing of newly ingested VA in the stomach, and 17 represents its processing in the intestine. Compartment 10 represents plasma retinyl ester in CM/CM remnants. Delay element 60 describes the time needed for the production of CM before their appearance in the plasma. Compartments 12, 13, 25, and 15 represent the uptake of plasma retinyl ester from CM/CM remnants into liver, lung, kidney, and carcass, respectively. Compartment 1 represents the plasma retinol pool. Compartments 2, 3, 4, 5, 6, and 7 represent VA pools in liver, lung, kidney, carcass, stomach, and intestine, respectively, that exchange VA with retinol in compartment 1. The asterisk represents the site of input of [^3^H]retinol and is also the site of input for dietary VA. IC(26) represents the newly ingested dose that enters the stomach.

After the organs were modeled individually, the plasma forcing function was released, and all data including tracer data for plasma and tissues (liver, lung, kidney, stomach, intestine, and carcass) were modeled simultaneously for both the control group and VARA group. Our proposed model is shown in **Fig. 5**. All of the compartment numbers, except compartment 26 and the delay element 60, are the same as in model 1 and have the same representation. Compartment 26 is the same as compartment 16 in model 1, representing the processing of newly ingested VA in the stomach, and 17 represents its processing in intestine. Compartment 10 represents plasma retinyl ester in CM/CM remnants. Delay element 60 describes the time needed for the production of CMs before their appearance in the plasma. Compartments 12, 13, 25, and 15 represent the uptake of plasma retinyl ester from CM/CM remnants into liver, lung, kidney, and carcass, respectively. The model indicates that, after being processed, plasma retinol, presumably as holo-RBP, is secreted from these tissues into compartment 1, the plasma retinol pool. Compartments 2, 3, 4, 5, 6, and 7 represent VA pools in liver, lung, kidney, carcass, stomach, and intestine, respectively, that exchange VA with retinol in compartment 1. The tracer data for plasma, liver, lung, kidney, carcass, stomach, and intestine were assigned to compartment 10 plus 1, 12 plus 2, 13 plus 3, 25 plus 4, 15 plus 5, 26 plus 6, and 17 plus 7, respectively. Liver (compartment 2) and carcass (compartment 5) were two sites of irreversible loss of retinol (9, 14, 15). A good fit to plasma and organ/tissue tracer data was obtained only when the output was from compartment 2 and compartment 5 (14).

To facilitate modeling of the full data set, some parameters were fixed based on results from model 1 or the plasma view model (5). Thus, L(12,10), L(13,10), L(25,10), L(15,10), and L(17,10), which represent the fraction of plasma retinyl ester in CM/CM remnants that is taken up/cleared by liver, lung, kidney, carcass, and intestine each day, respectively, were set up as fixed parameters whose values were the same as those in model 1. L(1,12), L(1,13), L(1,25), and L(1,15), which are the fractions of retinol secreted into plasma each day from liver, lung, kidney, and carcass, respectively, were fixed at the same value as L(0,12), L(0,13), L(0,25), and L(0,15) in model 1. L(2,1), L(3,1), L(4,1), L(5,1), L(6,1), and L(7,1) represent the fraction of plasma retinol that is transferred to liver, lung, kidney, carcass, stomach, and intestine each day, respectively. They were also set up as fixed values from model 1. L(1,2), L(1,3), L(1,4), L(1,5), L(1,6), and L(1,7) represent the fraction of retinol that recycles from corresponding extravascular tissues back to plasma per day. They were set up as adjustable parameters, and their initial values were based on the values of L(0,2), L(0,3), L(0,4), L(0,5), L(0,6), and L(0,7) in model 1. The setup of the L(I,J)s that describe the process of digestion and absorption in the model were based on the corresponding parts of our plasma-view model (5). Specifically, L(17,26), L(60,17), L(1,17), and L(0,17), which represent the fraction of retinol that enters the intestine from the stomach each day, the fraction of retinol that is incorporated into CM and secreted into lymph each day, the fraction of direct secretion of retinol from enterocytes into plasma each day, and the fraction of unabsorbed retinol per day, respectively, were fixed at values previously determined (5). L(0,2) and L(0,5) are adjustable L(I,J)s in model 2. They represent the fraction of irreversible loss of retinol from liver and from carcass per day, respectively. Several fixed L(I,J)s were made adjustable later in the modeling process to obtain a better fit.

The fit of the proposed model 2 to the data for both groups can be seen by comparing the observed data with the model-predicted values (supplementary Figs. II, III). **Table 4** shows kinetic parameters derived from model 2 for both groups. Adjustable parameters were well identified, with FSDs of 0.02–0.12 (not shown). Values for L(17,26) and L(60,17) in the two groups indicate that VARA accelerated the process of VA digestion and absorption in the gastrointestinal tract. L(0,17), the fraction of dose that was not absorbed, was very low in both groups, which indicates that the absorption efficiency of VA in pups is likely to be very high. VARA stimulated the uptake of CM retinyl ester by liver, lung, carcass, and intestine, as indicated by the values for L(12,10), L(13,10), L(15,10), and L(17,10) in the two groups. The secretion of retinol into plasma from liver and lung was dramatically inhibited by VARA, as evidenced by the values for L(1,12) and L(1,13). The unique L(I,J)s in model 2, L(0,2) and L(0,5), represent the fraction of irreversible loss of retinol from liver and from carcass, respectively. These values indicate that liver and carcass are the main sites of retinol disposal from the body in both groups.

**TABLE 4.** The fractional transfer coefficients predicted by the integrated model (model 2)

L(I,J)	Treatment Group	
	Oil	VARA
	Value (day^−1^)	
L(17,26)	4.26	13.9
L(0,17)	0.02	0.00
L(1,17)	0.55	0.00
L(60,17)	19.4	116
L(12,10)	100	223
L(1,12)	33.5	0.053
L(13,10)	10.0	27.0
L(1,13)	4.16	0.70
L(25,10)	3.38	1.41
L(1,25)	3.92	0.04
L(15,10)	89.9	439
L(1,15)	14.1	5.81
L(17,10)	0.00	327
L(2,1)	35.8	45.2
L(1,2)	1.46	1.18
L(0,2)	0.034	0.04
L(3,1)	0.22	0.14
L(1,3)	0.33	0.12
L(4,1)	3.40	3.10
L(1,4)	2.55	3.84
L(1,4), after dosing day 8	9.67	7.40
L(5,1)	5.97	7.25
L(1,5)	0.52	0.57
L(0,5)	0.18	0.66
L(6,1)	1.48	2.71
L(1,6)	5.00	7.37
L(1,6), after dosing day 8	7.47	6.46
L(7,1)	2.81	9.75
L(1,7)	0.65	1.07
L(1,7), after dosing day 8	2.39	2.07
IC(26)	0.30	0.35

The data are fractional transfer coefficients [L(I,J)s] or fraction of retinol in compartment J that is transferred to compartment I each day predicted by the integrated model (model 2). The model is shown in Fig. 5.

## DISCUSSION

By applying the forcing function option in WinSAAM, the whole-body system of VA kinetics in neonatal rats was uncoupled, and individual tissues were modeled separately first. As indicated by the results for the forcing function model (model 1), each organ/tissue was fit satisfactorily. The agreement between model-predicted and measured masses of retinol in liver and lung (Table 3) lend confidence in the generated model. An integrated model incorporating all tissues (model 2) was developed after the forcing function was released. Through these models, the trafficking and distribution of VA in neonatal rats, the contribution of different neonatal tissues to whole-body VA kinetics, and the effects of VARA supplementation on the kinetic behavior were evaluated.

From the tissue kinetic data, several interesting results were obtained. VARA increased the fraction of the ingested dose of retinol in liver at most time points and increased the fraction in lung in the first 2 days after dosing; the average peak fraction of the dose remaining in lung was 4 times higher in the VARA group than in the oil group. This effect of VARA agrees with mass data for lung reported previously (7). That is, VARA treatment apparently stimulated the uptake of [^3^H]retinol into neonatal liver and lung. It has been reported that VARA upregulates the mRNA expression of stimulated by retinoic acid gene 6, STRA6, which is implicated in the uptake of retinol (27).

Our model 1 predicts that tissues other than liver are important in taking up CM retinyl ester in neonates. The calculation of the percentage of plasma retinyl ester going to different tissues (Table 2) indicates that ∼52% of plasma retinyl esters was cleared by the liver, while as much as ∼40% was taken up by the carcass. The role of extrahepatic tissues in taking up plasma retinyl ester from CM in neonatal rats was more prominent in VARA-treated pups, in which liver took up only 22% of plasma retinyl ester while the remaining 78% was taken up by extrahepatic tissues. Because neonates are born with a low VA level but presumably have a high requirement for it, we speculate that the uptake of dietary retinyl ester by extrahepatic tissues is an adaptive mechanism in neonates to make VA more readily available for utilization in tissues. CM retinyl ester might serve as a direct precursor for retinol and the synthesis of RA in tissues where RA is needed. As mentioned, previously it was reported that the level of LPL expression in skeletal muscle, heart, and adipose tissue influences the amount of [^3^H]retinoid taken up from CM and/or their remnants. It has also been shown that LPL is able to catalyze the hydrolysis of retinyl ester in CM in adult rats and, through the process of hydrolysis, may facilitate uptake of retinoid by these tissues (12, 28). Although it is still not clear how CM retinyl ester is taken up by neonatal extrahepatic tissues, it is reasonable to hypothesize that LPL contributes as in adults. It was reported that LPL activity in rat lung, skeletal muscle, heart muscle, and brown adipose tissue emerged substantially during the first 24 h after birth, and the enzyme activity in brown adipose tissue and skeletal muscle was highest during suckling compared with other periods of life (29). The VARA treatment, especially the RA component, might stimulate the uptake of CM retinyl ester by extrahepatic tissues by inducing LPL expression and/or its activity in these tissues. More than one mechanism may exist to mediate the uptake of CM retinyl ester in neonates. Future experiments focusing on CM retinyl ester could be designed to confirm the findings predicted by these models, as well as to explore the mechanisms involved and the effects of VARA on them. For example, lymph containing CM labeled with [^3^H]retinyl ester (30) could be injected into neonatal rats for investigation of CM metabolism.

Our models indicate that VARA supplementation altered the trafficking and distribution of the tracer dose compared with results in unsupplemented neonates. VARA supplementation stimulated the uptake of plasma retinyl ester in CM/CM remnants by liver, lung, carcass, and intestine [L(12,10), L(13,10), L(15,10), and L(17,10), respectively, Tables 1 and 4]. VARA decreased the secretion of retinol, presumably as holo-RBP because nearly all plasma retinol is present bound to RBP, into plasma from liver, lung, and kidney [L(1,12), L(1,13), and L(1,25), respectively, in Table 4)]. In the VARA group, the values for L(1,12), L(1,13), and L(1,25), which represent the fraction of retinol secreted into plasma from liver, lung, and kidney, respectively, are all close to zero. These findings from the model indicate that VARA stimulated the uptake of plasma retinyl ester into tissues and that more VA stayed in tissues, perhaps for storage, instead of being secreted into plasma. In addition, the model predicts that in VARA-treated pups very little retinol as holo-RBP was secreted into plasma from liver (most was from carcass). The model also predicts that VARA increased the fraction of retinol that was transferred from plasma holo-RBP into carcass and decreased the fraction of retinol turnover out of the liver each day, indicated by L(5,1) and L(0,2) of model 1, respectively.

The model allowed the distribution of VA in neonatal rats to be estimated from the calculated percentage of plasma VA uptake and turnover into tissues (Table 2), and the model-predicted retinol pool sizes in different tissues in the period prior to 2 days after dosing in oil-treated pups (Table 3). Of the plasma retinol turnover into different tissues (Table 2), ∼76% went to liver, ∼13% went to the carcass, and 4.8% went to the intestine in control pups. That is, the majority went to liver, as has been reported for adult rats (9). VARA supplementation increased the percentage that went to the intestine to ∼21% and the percentages that went to liver and carcass to 50% and 15%, respectively. Thus, VARA supplementation altered not only the amount but the distribution of the traced mass in the neonatal body. We hypothesize from the results of the model calculations and prediction (Table 3) that the rank order of VA distribution in tissues in oil-treated neonatal rats in the steady state is liver > carcass (mainly skin, bone, adipose tissue, and brain) > intestine > kidney > lung, stomach. The model predicted that ∼51% of whole-body VA was extrahepatic in neonatal (pnd 4–6) rats with marginal VA status [as shown in (5)]. A previous report showed that 44% of whole-body VA was extrahepatic in adult rats with marginal VA status (9).

Based on our modeling results, we conclude that carcass plays an important role in VA kinetics in neonatal rats. Approximately 40% of plasma retinyl ester in CM was taken up by carcass, and ∼15% of plasma retinol turnover went to the carcass. The models predicted that in VARA-treated pups, the carcass, rather than the liver, was the most important source of the retinol secreted into plasma. Presently, which tissues of the carcass are most important is not known. However, we speculate that skin may play an important role, as it is a major organ in terms of mass and it is known both that a fundamental function of VA is to control normal epithelial differentiation (31) and that VA and RA are important for skin development (32). Neonates have a higher surface area-to-volume ratio than adults and therefore they may have a higher requirement for VA for skin development and maintenance. VA is also important for skeletal development (33). Bone and skeletal muscle in neonates may take up newly ingested VA for utilization. In adult rabbits, one-half to one-third of VA-labeled CMs were removed by bone marrow, and CM may be a significant source of VA for this tissue (34). It has been reported that adipose tissue in adult rats may also acquire newly absorbed VA during lipolysis of CM by LPL (12). Pups in our experiment did not have much white adipose fat, which was hardly observable before pnd 10. However, brown adipose tissue, which is known to be prominent in newborns (35), may play a role in the VA kinetics of the analyzed carcass. Brown adipose tissue makes up about 5% of the body mass in human neonates (35) and about 0.5–1% in rat neonates (29). The tissue is of importance for thermogenesis in neonates, who are more susceptible to cold than adults (35). In future studies, these tissues can be isolated and collected for detailed investigation and treated as separate compartments in the modeling process.

Results for the intestine are also interesting. To obtain a good fit of the data, it was necessary to include the process of uptake of CM retinyl ester to the intestine, which is represented by L(17,10). Although L(17,10) was low in the control group, the VARA treatment stimulated the uptake dramatically, with the percentage uptake of plasma retinyl ester by intestine being estimated as ∼30%. VARA also stimulated plasma retinol turnover into the intestine. The treatment appears to have a short-term effect of accelerating VA digestion and absorption in the gastrointestinal tract, and it might also influence the longer-term kinetics of retinol flux between plasma and intestinal pools. A previous report showed that VA deficiency in adult rats inhibited intestinal adaptation following partial small bowel resection (36). It was also found that RA administered either iv or as a pellet had significant trophic effects in bowel-resected rats (37). Our model-predicted effects of VARA on stimulating the uptake of plasma retinyl ester and plasma retinol turnover into the intestine may be related to a positive influence of VA on the intestinal maturation of neonatal rats. Future experiments suggested by these models may provide new insights into neonatal VA intestinal physiology. When developing the model for the intestine, we also considered the process of enterohepatic circulation, as it is known that VA in the liver may be converted into water soluble metabolites that are excreted in bile (38). We thus hypothesized that some proportion of VA might circulate back to the intestine through such a process in neonates. However, the addition of compartments to represent this process did not improve the fit of the model, and so it was not included. This may indicate that the amount of VA in the enterohepatic circulation in neonatal rats is not significant.

A comment is worthwhile regarding the strengths of models 1 and 2. Some difficulties were encountered in the development of the integrated model, model 2, when we released the forcing function applied in model 1. Because the [^3^H]retinol tracer was given orally, tracer responses in tissues were much more complex than those in previous studies in which tracer was injected into plasma in adult rats (9, 15; see also Ref. 5 for discussion). The complexity of responses was further increased because the experimental subjects were neonates, which inherently are under metabolic nonsteady state due to rapid growth. Modeling tracer data for plasma and all of the tissues simultaneously was therefore difficult. Although model 2 was successfully developed and satisfactorily fit, as reflected by the sum of squares and visual examination of the curves, it is still not as well identified as model 1, which has a better fit of the tissue tracer data. As a result, we relied more on model 1 than model 2 for information about the contribution of the various organs to whole-body VA kinetics. However, this does not mean that model 2 is meaningless. It could be a starting point in future studies for the development of compartmental models of neonatal VA kinetics under other treatment conditions.

In conclusion, by applying the forcing function option in WinSAAM, each tissue of the neonatal rat was fit satisfactorily, and then an integrated model incorporating all tissues was successfully developed after the forcing function was released. Our models predicted several features of neonatal VA metabolism that were not anticipated, including that neonatal extrahepatic tissues play an important role in clearing CM retinyl ester, especially in VARA-treated pups, and that a significant amount of extrahepatic VA is present in neonatal rats. Additionally, VARA supplementation increased the uptake of CM retinyl ester by lung, carcass, and intestine, and decreased the release of retinol, that had been cleared by liver and lung as CM retinyl ester, into plasma. VARA supplementation was also predicted to stimulate the uptake of plasma retinol into the carcass and to decrease retinol turnover out of the liver. The whole-body models developed in this study suggest several additional hypotheses, which can be tested in future studies in the rat or in other neonatal models.

## Supplementary Material

Supplemental Data
