# Supplementary material for: Compartmental modeling of whole-body vitamin A kinetics in unsupplemented and vitamin A-retinoic acid-supplemented neonatal rats
Source: J Lipid Res. 2014 Aug;55(8):1738–49. doi: 10.1194/jlr.M050518 (PMC4109768; doi:10.1194/jlr.M050518)
Supplement: Supplemental Data [file supp_55_8_1738__index.html]

Compartmental modeling of whole-body vitamin A kinetics in unsupplemented and vitamin A-retinoic acid supplemented neonatal rats — Compartmental modeling of whole-body vitamin A kinetics in unsupplemented and vitamin A-retinoic acid-supplemented neonatal rats — Supplemental Data 

# Compartmental modeling of whole-body vitamin A kinetics in unsupplemented and vitamin A-retinoic acid-supplemented neonatal rats

## Supplemental Data

**Files in this Data Supplement:**

- Fig. S1 - Supplemental figure S1.
- Fig. S2 - Supplemental figure S2.
- Fig. S3 - Supplementary figure S3.
