# Supplementary material for: Compartmental modeling of whole-body vitamin A kinetics in unsupplemented and vitamin A-retinoic acid-supplemented neonatal rats
Source: J Lipid Res. 2014 Aug;55(8):1738–49. doi: 10.1194/jlr.M050518 (PMC4109768; doi:10.1194/jlr.M050518)
Supplement: Supplemental Data [file supp_M050518_jlr.M050518-1.pdf]

A

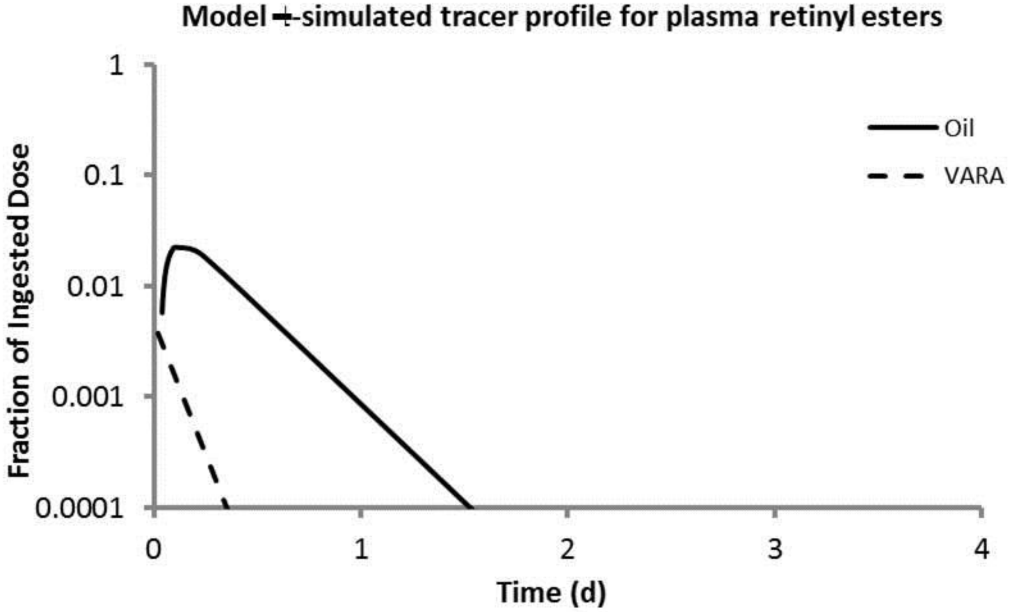

B

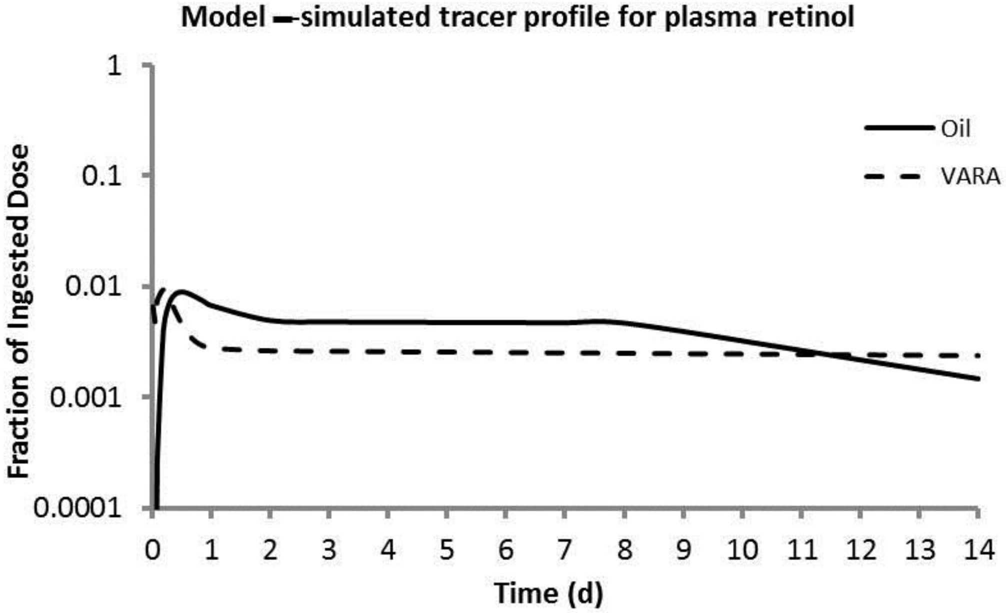

Figure S1. Plasma view model-simulated tracer response profiles for plasma chylomicron (CM) retinyl ester (compartment 10 in the plasma view model; panel A) and plasma retinol (compartment 5 in the plasma view model; panel B) for both the control group and VARA-treated group.
