# Supplementary material for: Compartmental modeling of whole-body vitamin A kinetics in unsupplemented and vitamin A-retinoic acid-supplemented neonatal rats
Source: J Lipid Res. 2014 Aug;55(8):1738–49. doi: 10.1194/jlr.M050518 (PMC4109768; doi:10.1194/jlr.M050518)
Supplement: Supplemental Data [file supp_M050518_jlr.M050518-3.pdf]

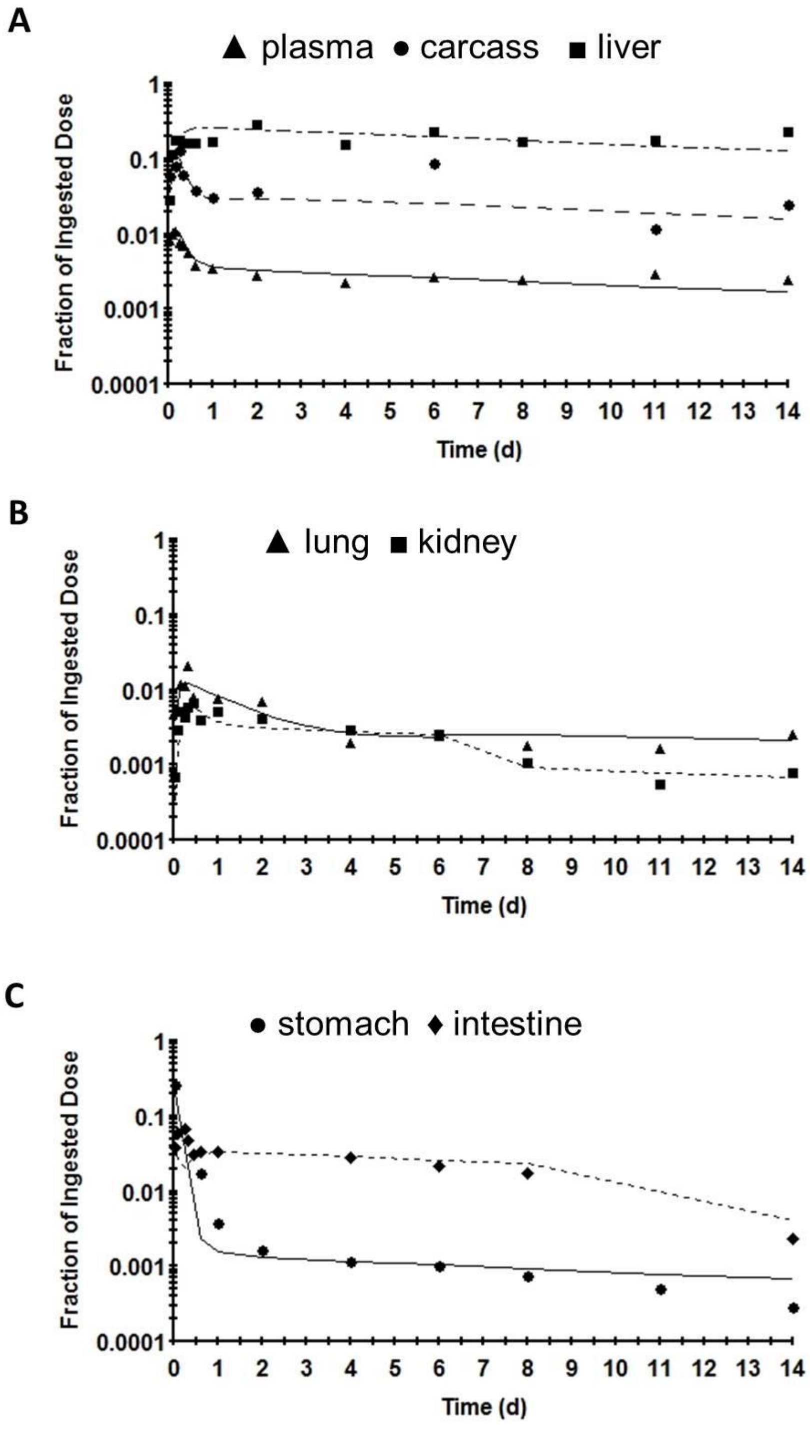

Figure S3. Mean observed (symbols) and model 2-predicted (lines) fraction of administered dose in plasma, liver, and carcass (A); lung and kidney (B); and stomach and intestine (C) vs. time (d) after administration of [^{3}H]retinol in VARA to neonatal rats. Each point represents the mean of n=3 pups.
